# Calcinosis Cutis as an Unsuspecting Complication of Hyperkalemia Treatment

**DOI:** 10.7759/cureus.17018

**Published:** 2021-08-09

**Authors:** Lauren N Gresham, Jwan Alallaf, Jignesh Shah

**Affiliations:** 1 Dermatology, University of Missouri Kansas City School of Medicine, Kansas City, USA; 2 Pathology, University of Missouri Kansas City School of Medicine, Kansas City, USA; 3 Nephrology, University of Missouri Kansas City School of Medicine, Kansas City, USA

**Keywords:** calcinosis cutis, calcium gluconate, bullous dermatoses, extravasation, hyperkalemia

## Abstract

Calcinosis cutis is a known but rare complication from the extravasation of intravenous calcium preparations. Calcium gluconate is a commonly used medication to prevent cardiac arrhythmias in the setting of hyperkalemia and cardiac arrest during resuscitation and life support. Extravasation of calcium gluconate may result in skin necrosis and a bullous reaction in its most severe form, which should be promptly recognized so that treatment can be provided. Pediatric patients are more susceptible to this caustic effect while cases in adults are rare. We report the case of a patient who developed bullous skin lesions with skin necrosis and eschar formation after receiving intravenous calcium gluconate for the treatment of hyperkalemia. The patient required an extensive hospital stay and multiple surgical interventions. This case demonstrates that common medications such as calcium gluconate can lead to significant adverse effects that can be mitigated with proper administration and appropriate education about adverse events.

## Introduction

Intravenous calcium gluconate is a medication used to prevent cardiac arrhythmias in the setting of hyperkalemia and cardiac arrest during resuscitation and life support [1]. It antagonizes the action of potassium, restores the transmembrane voltage gradient, and stabilizes the cardiac cell membrane [1]. Calcium gluconate is a relatively safe medication and is commonly used in medical wards and emergency rooms across the world [1]. Although rare, the most concerning adverse effect is extravasation into the surrounding tissue [1]. Calcium induces local vasoconstriction leading to tissue necrosis and deposits in the subcutaneous tissue which causes local calcifications, called calcinosis cutis [1,2]. A few hours after infusion, patients present with rapid swelling and erythema of the skin surrounding the intravenous site and may have skin necrosis [2]. We report the case of a patient who developed bullous skin lesions with necrosis and eschar formation a few hours after receiving calcium gluconate for the treatment of hyperkalemia.

## Case presentation

**Figure 1:**
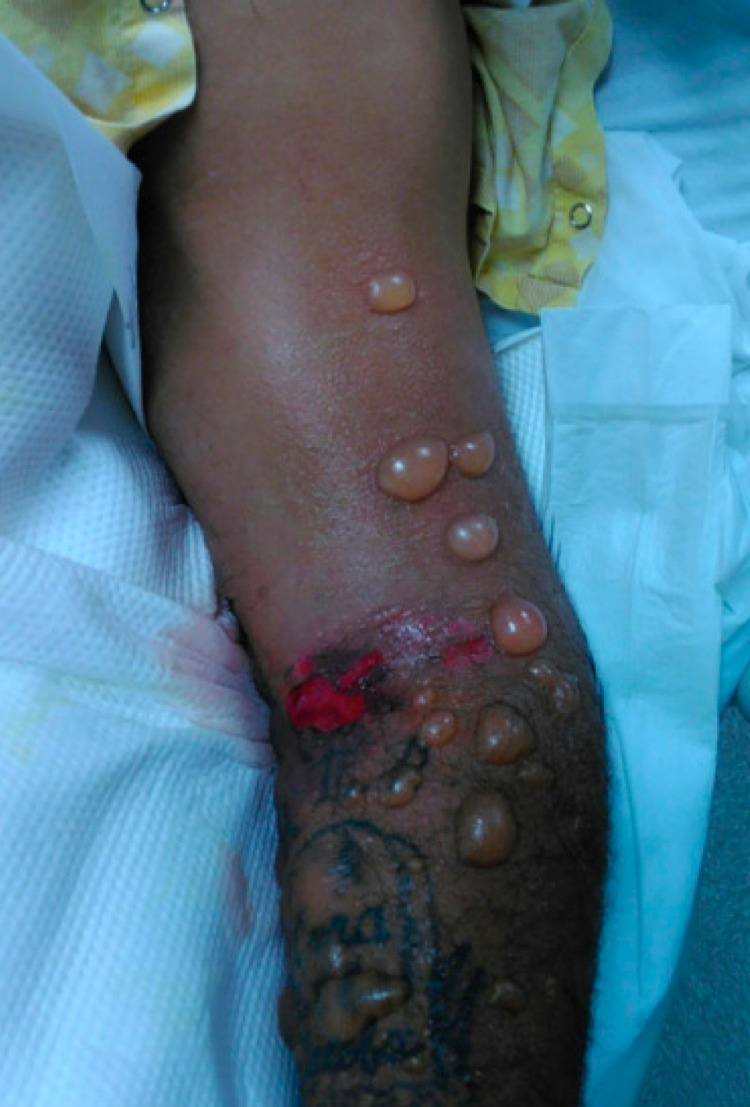
Initial bullous reaction. Central necrotic lesion with surrounding bullous lesions located on the patient’s left upper extremity.

**Figure 2:**
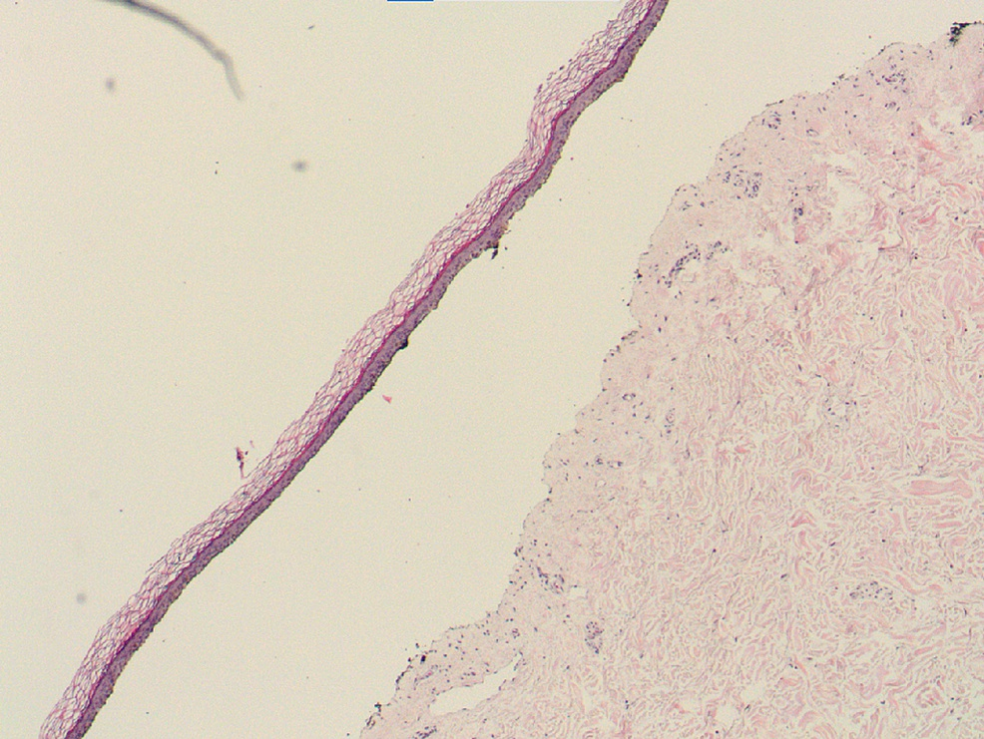
Punch biopsy. Hematoxylin and eosin stain of left upper extremity punch biopsy showing pauci-inflammatory subepidermal blister.

A 35-year-old male with a history of seizure disorder, traumatic brain injury, and cannabis abuse presented to the emergency department with multiple gunshot wounds to the lower abdomen and left hip. The patient was emergently taken to the operating room for an exploratory laparotomy. He required a small bowel resection with re-anastomosis and was found to have multiple enterotomies to the mid-jejunum, a defect to the greater omentum, and a small left retroperitoneal hematoma, which were repaired appropriately. The patient required significant colloid and crystalloid resuscitation for hypovolemic shock. Consequently, he developed hyperkalemia from transfusion protocol and an acute kidney injury from shock and renal hypoperfusion. The patient was treated conservatively with calcium gluconate, insulin, and intravenous fluids. He was later treated with loop diuretics resulting in the resolution of his hyperkalemia and acute kidney injury. He received two doses of 1 g of calcium gluconate intravenously in the left antecubital fossa. A few hours later, he developed a necrotic lesion with numerous surrounding bullous lesions (Figure 1). Compartment syndrome and deep vein thrombosis were ruled out. Initially, cellulitis was suspected but the bullae led to suspicion for immunological disease. A punch biopsy of the skin was performed which showed pauci-inflammatory subepidermal blisters (Figure 2). Direct immunofluorescence was negative for immune deposits, which ruled out autoimmune bullous disease. This finding was attributed to calcium gluconate extravasation causing local calcinosis cutis.

**Figure 3:**
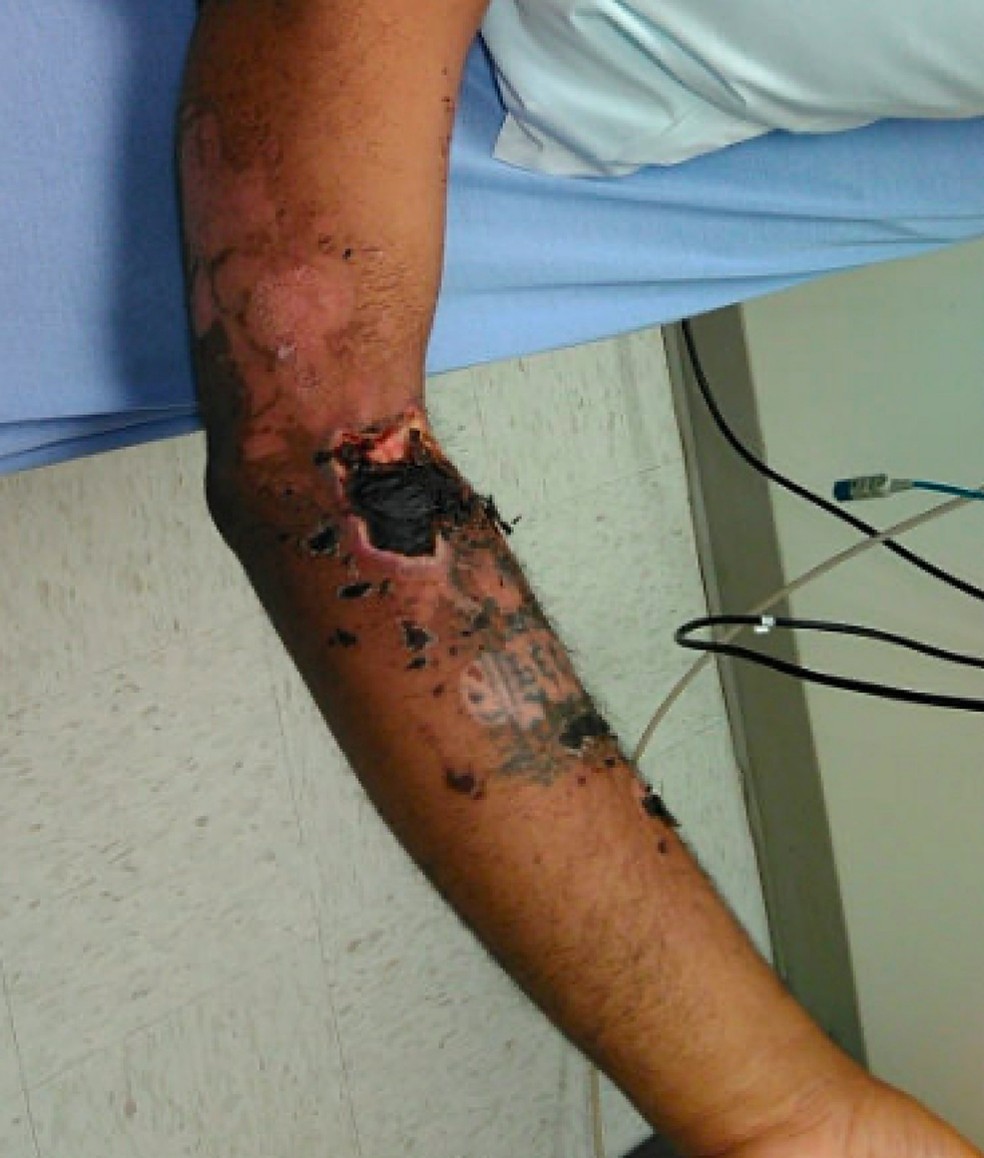
Wound progression. Progression of left upper extremity wound with slough and eschar formation.

**Figure 4:**
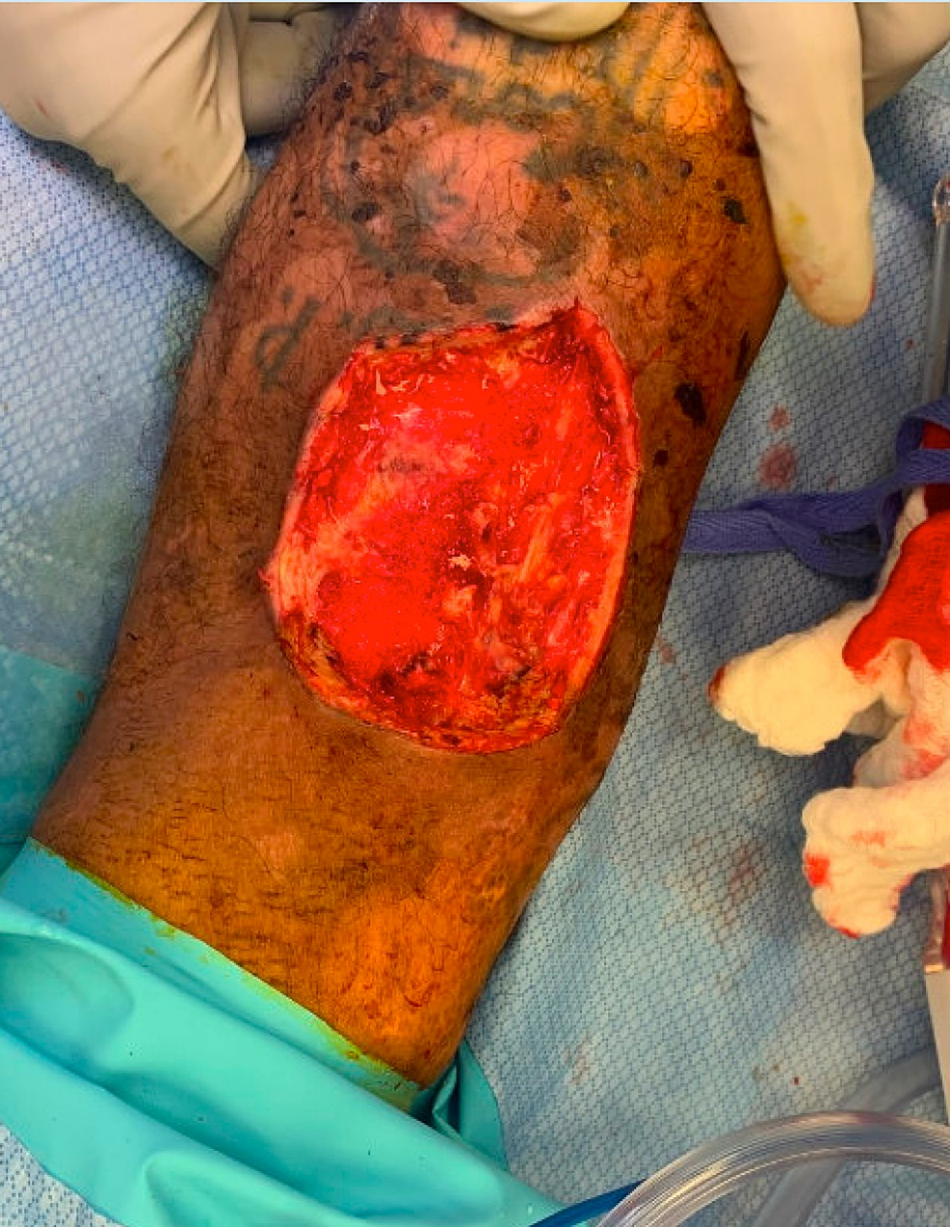
Debridement. Left upper extremity wound after debridement in the operating room.

**Figure 5:**
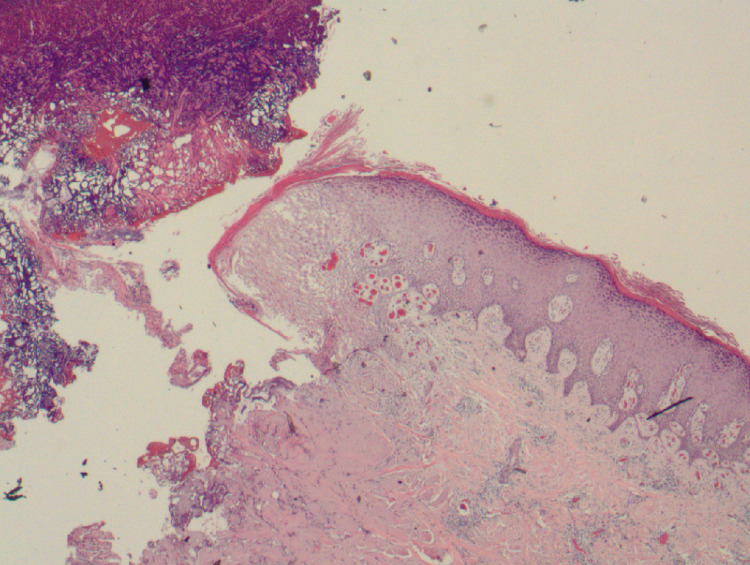
Excisional biopsy. Hematoxylin and eosin stain of left upper extremity excisional biopsy demonstrating focal gangrenous necrosis and mixed acute and chronic inflammation.

The patient required wound care and debridement at the lesion. However, his course became complicated as he was discharged from the hospital when his other ailments improved and he was nonadherent with his therapy at home. He presented to the primary care clinic three weeks later with wound progression and slough formation with eschar (Figure 3). He required another debridement and was reassigned to the wound care clinic (Figure 4). An excisional skin biopsy was performed which showed gangrenous necrosis with signs of acute and chronic inflammation (Figure 5). The wound eventually improved, but this adverse reaction to calcium gluconate led to a more complicated and lengthy hospital course than predicted by his original admission for a gunshot wound.

## Discussion

Extravasation of calcium-containing medications may lead to iatrogenic calcinosis cutis, and, in rare cases, may lead to a bullous reaction of the skin [2,3]. The pathogenesis of bullous reactions after intravenous calcium gluconate extravasation is not well-understood and cannot be histopathologically differentiated from other nonimmune-mediated bullous disorders [2]. However, the onset of this patient’s bullous skin reaction soon after intravenous calcium gluconate infusion and localization around the site of infusion indicate a causal relationship which may be explained by the extravasation of calcium gluconate. Although extravasation of other medications has been associated with a bullous skin eruption, only two cases of such reactions caused by calcium gluconate have been reported in the literature [2,3]. Overall, the incidence of adverse events due to extravasation of calcium gluconate is poorly understood as such events are usually reported as single cases, most often in infants receiving treatment for hypocalcemia [4]. An analysis of current cases of iatrogenic calcinosis cutis found that 55% of cases occurred in neonates aged up to one month and 20% occurred in adults [4]. Adverse reactions to extravasation are more common with calcium chloride than calcium gluconate, likely because calcium chloride dissociates more extensively leading to a higher risk for precipitation [5].

Although severe reactions to calcium gluconate extravasation are rare, clinicians need to be aware of all potential adverse effects so that they can be promptly recognized and treated effectively [1]. When extravasation is noted immediately, the infusion should be discontinued and the extravasated fluid should be aspirated from the surrounding tissue [1]. In cases of early extravasation, hyaluronidase or triamcinolone acetonide may be administered intradermally; however, there is little evidence for the effectiveness of these therapies [1,5]. Recently, sodium thiosulfate has shown efficacy in two cases of calcinosis cutis [6,7]. Otherwise, management is supportive and includes cold and dry compresses, elevation of the extremity, local wound care, and surgical debridement, as seen in our patient [1,5]. Prevention of extravasation with proper infusion techniques is the most important mitigating factor [5]. The risk of extravasation can be reduced by optimal vein selection, regular assessment of established intravenous sites, using a central line for administration, and administration of a diluted solution [5,7]. Early recognition of extravasation is also vital and involves a team-based approach with close monitoring by the nursing staff [1].

## Conclusions

A rare complication of intravenous calcium gluconate infusion is extravasation, which may result in calcinosis cutis, necrosis, and a bullous reaction in its most severe form. Although a bullous reaction after an intravenous calcium gluconate infusion is extremely rare, clinicians should be aware of this potential adverse effect. Proper administration of calcium gluconate and other risk reduction strategies can reduce the potential for such events, preventing prolonged and complicated hospital courses for patients. More thorough reporting of this complication and understanding of the pathophysiology are needed to improve patient management when such events occur.
